# A longitudinal transcriptomic analysis from unfed to post-engorgement midguts of adult female *Ixodes scapularis*

**DOI:** 10.1038/s41598-023-38207-5

**Published:** 2023-07-13

**Authors:** Stephen Lu, Larissa A. Martins, Jan Kotál, José M. C. Ribeiro, Lucas Tirloni

**Affiliations:** 1 Vector Biology Section, Laboratory of Malaria and Vector Research, National Institute of Allergy and Infectious Diseases, Bethesda, MD USA; 2 Tick-Pathogen Transmission Unit, Laboratory of Bacteriology, National Institute of Allergy and Infectious Diseases, Hamilton, MT USA; 3 Laboratory of Persistent Viral Diseases, Neuroimmunology Unit, National Institute of Allergy and Infectious Diseases, National Institutes of Health, Hamilton, MT USA

**Keywords:** Molecular biology, Transcriptomics

## Abstract

The hematophagy behavior has evolved independently several times within the Arthropoda phylum. Interestingly, the process of acquiring a blood meal in ticks is considerably distinct from that observed in other blood-feeding arthropods. Instead of taking seconds to minutes to complete a blood meal, an adult female *Ixodes scapularis* tick can remain attached to its host for numerous days. During this extended feeding period, the tick undergoes drastic morphological changes. It is well established that the tick midgut plays a pivotal role not only in blood meal digestion but also in pathogen acquisition and transmission. However, our understanding of the underlying molecular mechanisms involved in these events remains limited. To expedite tick research, we conducted a comprehensive longitudinal RNA-sequencing of the tick midgut before, during, and after feeding. By collecting ticks in different feeding stages (unfed, slow feeding, rapid feeding, and early post-detached), we obtained a comprehensive overview of the transcripts present in each stage and the dynamic transcriptional changes that occur between them. This provides valuable insights into tick physiology. Additionally, through unsupervised clustering, we identified transcripts with similar patterns and stage-specific sequences. These findings serve as a foundation for selecting targets in the development of anti-tick control strategies and facilitate a better understanding of how blood feeding and pathogen infection impact tick physiology.

## Introduction

Ticks are exclusive blood-feeding arthropods that can serve as vectors for multiple pathogens relevant to human and veterinary health^1^. Currently, *Ixodes scapularis* is the primary vector of *Borrelia burgdorferi*, the causative agent of Lyme disease in the United States^2^. Since effective vaccines against most tick-borne pathogens are lacking, the prevention of human tick-borne diseases relies on mitigating tick bites. Tick control primarily relies on acaricides, which has several drawbacks, prompting the exploration of alternative control methods. Immunological control has shown promise as a viable alternative, but identifying key antigens remains a challenge^3^.

It is estimated that the blood-feeding behavior evolved independently multiple times throughout evolution, with hematophagy appearing in ticks over 100 million years ago^4^. Notably, ticks have adopted a somewhat unique strategy for obtaining their blood meal compared to other hematophagous arthropods. While most species engage in rapid feeding, completing their blood meal within seconds to minutes, hard ticks like *I. scapularis* remain attached to their host for multiple days. Ticks obtain a blood meal by cutting the skin and small blood vessels, ingesting the blood from a feeding pool. The feeding process occurs over several days and comprises three stages: preparatory feeding, where the tick attaches itself to the host's skin and creates a feeding pool; slow feeding, where the tick consumes moderate amounts of blood, starts transmitting pathogens, and develops and prepares new tissue for rapid feeding, where it feeds to repletion^5^.

Blood meal digestion in ticks also differs from other hematophagous vectors. Instead of taking place in the midgut lumen surrounded by a peritrophic matrix, ticks uptake hemoglobin through digestive cells^6^ and cleave it intracellularly, mainly using aspartic and cysteine peptidases^7,8^. Additionally, the tick midgut serves as the primary entry site for pathogens, where key interactions necessary for pathogen survival and proliferation occur^9,10^. Therefore, targeting relevant physiological processes in the midgut could be a viable strategy for developing effective anti-tick and/or pathogen-blocking control methods^11–13^. Several groups have explored the midgut contents of multiple tick species^14–19^. However, most of these studies focused on limited time points or the early stages of feeding, offering an incomplete picture of the tick midgut as feeding progresses.

In this study, we investigated the transcriptional changes that occur in the midgut of adult female *I. scapularis* ticks as feeding progresses. Uniquely, instead of grouping the different feeding stages by “days of feeding”, ticks were grouped based on their average weight, representing the unfed (UF), slow-feeding (G1, G2, G3, and G4), rapid-feeding (G5 and G6), and early post-detachment (24-, 48-, and 72-h post-detachment) phases. This study not only enhances our understanding of how blood feeding impacts tick midgut physiology but also aids in identifying targets for the development of anti-tick and/or pathogen-blocking control strategies.

## Materials and methods

### Ethics statement

Animal experiments were conducted in accordance with the guidelines of the National Institutes of Health on protocols approved by the Rocky Mountain Laboratories Animal Care and Use Committee (Protocol: 2020-065). The Rocky Mountain Veterinary Branch is accredited by the International Association for Assessment and Accreditation of Laboratory Animal Care (AAALAC).

### Tick rearing and midgut dissection

Specific pathogen-free (*Borrelia burgdorferi *and *Anaplasma phagocytophilum*) *Ixodes scapularis* ticks were purchased from the tick rearing facility at Oklahoma State University. Unfed ticks were maintained at 21 °C and 80–90% relative humidity before infestation. One day prior to feeding, female ticks were paired with males to mate. Adult ticks used for midgut extraction were restricted to feed onto the outer part of the ear of four naïve female New Zealand White rabbits with orthopedic stockinet glued. A total of 20 adult females and 20 males (40 ticks per ear, 80 ticks per animal) were placed into the tick containment apparatus and allowed to attach. Ticks were collected and organized into 10 biological conditions based on their average weight or hours post-detachment. For each biological condition, we obtained three independent samples and each sample consisted of the pooled midgut of ticks. To group ticks by a blood feeding index, partially fed ticks were collected from the host during the feeding, selected by their engorgement size, and sorted by their average weight in biological triplicates: group unfed (UF, n = 5 ticks per replicate; average weight:1.6 ± 0.19 mg), G1 (n = 5 ticks per replicate; average weight 2.9 ± 0.64 mg), G2 (n = 5 ticks per replicate; average weight 7.4 ± 0.93 mg), G3 (n = 3 ticks per replicate; average weight 15.9 ± 1.81 mg), G4 (n = 3 ticks per replicate; average weight 24.6 ± 3.17 mg), G5 (n = 3 ticks per replicate; average weight 44.5 ± 6.25 mg) and G6 (n = 2 ticks per replicate; average weight 105 ± 17.45 mg). Fully engorged ticks were dissected at 24- (n = 3 ticks per replicate; weight not recorded), 48- (n = 3 ticks per replicate; weight not recorded), and 72-h (n = 3 ticks per replicate; weight not recorded) post-detachment (hpd). After removal from the host, ticks were rinsed with bleach 1%, nuclease-free water, and ethanol 70%, following a last rinsing with nuclease-free water. Ticks were dissected within two hours after removal from the host. Tick midguts (MGs) were dissected in a fresh ice-cold nuclease-free phosphate-buffered saline (PBS), pH 7.4 (Invitrogen). After dissection, MGs were washed gently in a fresh nuclease-free PBS, pH 7.4 containing RNAse inhibitor (RNaseOUT, Thermo Fisher Scientific). After washing, dissected MGs were stored immediately in RNAlater (Invitrogen) until total RNA extraction.

### Library preparation, sequencing, and data analysis

Total RNA was isolated using the AllPrep DNA/RNA/Protein mini kit (QIAGEN) according to the manufacturer's instructions. RNA integrity and quantity were assessed using a 4200 TapeStation system (Agilent Technologies). The Illumina libraries were constructed using the NEBNextUltraTM II (Directional) RNA with polyA selection library prep kit and sequencing was performed in an Illumina Novaseq 6000 DNA sequencer. The quality of raw Illumina reads was checked using the FastQC tool (https://www.bioinformatics.babraham.ac.uk/projects/fastqc/) and trimmed from their adaptor and any low-quality sequence (Q < 20) with TrimGalore (https://github.com/FelixKrueger/TrimGalore). After trimming, reads were mapped to the *I. scapularis* genome (GCF_016920785.2, RefSeq release 103)^20^ with the RSEM tool^21^ and transcripts that presented an average of transcripts per million (TPM) ≥ 5 in at least one biological condition were used for further downstream analysis. Functional annotation of the selected coding DNA sequences (CDS) was performed by an *in-house* program that scans a vocabulary of ~ 400 words and their order of appearance in the protein matches from BLASTp/rpsBLAST results against different databases (Transcriptome Shotgun Assembly, subset of the Non-Redundant, Refseq-invertebrate, Refseq-vertebrate, Refseq-protozoa, *I. scapularis* genome, UNIPROT, CDD, SMART, MEROPS and PFAM), including their percent identities and coverage. The final annotated CDS were exported as a hyperlinked excel and is currently available for download (Supplementary File 1).

### Statistical analysis

The multidimensional plot and the differential expression analysis were carried out with the edgeR package^22^ for R^23^. Transcripts were considered differentially expressed when a foldchange of at least ± 4 and the false discovery rate (FDR) less than 0.05 were obtained. The heatmap plot was generated with the pheatmap package using the TPM values as percentages and the volcano plot was generated with the ggplot2 package for R. Unsupervised clustering of the filtered CDS was performed with the Expander tool using the CLICK method^24^.

## Results and discussion

### Overview of the temporal transcriptome analysis of *I. scapularis* midgut

**Figure 1:**
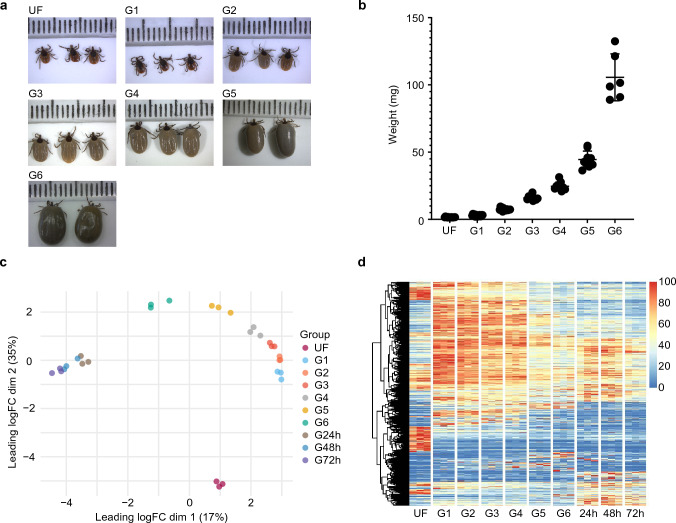
Overview of the transcriptome profile of *I. scapularis* midgut at different feeding stages. (**a**) Visual representation of unfed and partially fed *I. scapularis* adult females collected at different feeding stages and grouped by their (**b**) average weight (± standard error of the mean).This plot was generated using GraphPad Prism 8.0. Each dot on the scale in Figure 1A represents 1 mm. (**c**) PCA plot of the transcripts with TPM ≥ 5 in at least one of the biological conditions. (**d**) Heatmap plot using the normalized TPM value of each transcript. UF represents the unfed ticks, G1–G6 represents ticks with different average weights 2.9 mg (G1), 7.4 mg (G2), 15.9 mg (G3), 24.6 mg (G4), 44.5 mg (G5), and 105 mg (G6). 24 h, 48 h, and 72 h represent ticks collected 24, 48, or 72 h post-detachment from the host. PCA plot was generated using the edgeR (3.42.4)^22^ and ggplot2 (3.4.2) packages for R^23^.

Illumina sequencing of 30 libraries from *I. scapularis* midgut at different feeding stages (Fig. 1a and b) resulted in 1,644,351,120 high-quality reads. Mapping the trimmed reads to the current transcripts annotated in the *I. scapularis* genome yielded similar alignment rates across all libraries (63.92% ± 1.6%). For functional annotation and differential expression analysis, we extracted sequences that showed an average TPM ≥ 5 in at least one of the 10 biological conditions, resulting in 10,080 CDS and 479 sequences currently annotated as non-coding RNA (ncRNA). The final sequences and their functional annotation were exported to a Windows-compatible hyperlinked Excel file, which is currently available for download (Supplementary File 1). Additionally, the Benchmarking Universal Single-Copy Orthologs (BUSCO) analysis with the filtered transcripts resulted in a completeness of 86.0% (71.4% single and 14.6% duplicated), with 0.1% fragmented and 13.9% missing. The consistent alignment rates and the completeness of the current dataset indicate the absence of major bias in our libraries and reflect the overall quality of our samples.

Initial data exploration through dimension analysis revealed that all biological replicates clustered within their respective biological conditions (Fig. 1c), indicating that grouping ticks by weight provides a more precise estimation of the current tick feeding stage compared to the commonly used "days of feeding" variable^25^. Additionally, the PCA plot indicates that the unfed (UF) group exhibits the most distinct transcriptional profile among all biological conditions, while the midgut of recently detached ticks (24-, 48-, and 72-hpd) appears to have a generally similar profile, as little separation was observed between the three groups. Previous transcriptome studies focused on the midgut of ticks at limited time points^14–16^ or specifically focused on the early feeding stages^26^, providing only a partial understanding of the changes occurring during feeding progression. By collecting ticks at multiple feeding stages (i.e., unfed, slow-feeding, rapid-feeding, and early post-detachment), our data offers a higher resolution of the transcriptional modulation in the tick midgut. The heatmap plot (Fig. 1d), utilizing normalized TPM values of each transcript, provides an overview of such modulation. While several transcripts were found to be almost exclusively present in unfed ticks, others were upregulated during the early feeding stages (G1–G4) and gradually decreased as the tick became engorged and detached from its host. Moreover, the heatmap plot suggests the presence of four main transcriptional profiles that can be assigned to four biological states of the tick midgut: (1) unfed, (2) slow-feeding phase (G1-G4), (3) rapid-feeding phase (G5-G6), and (4) early post-detachment (24–72-hpd).

**Figure 2:**
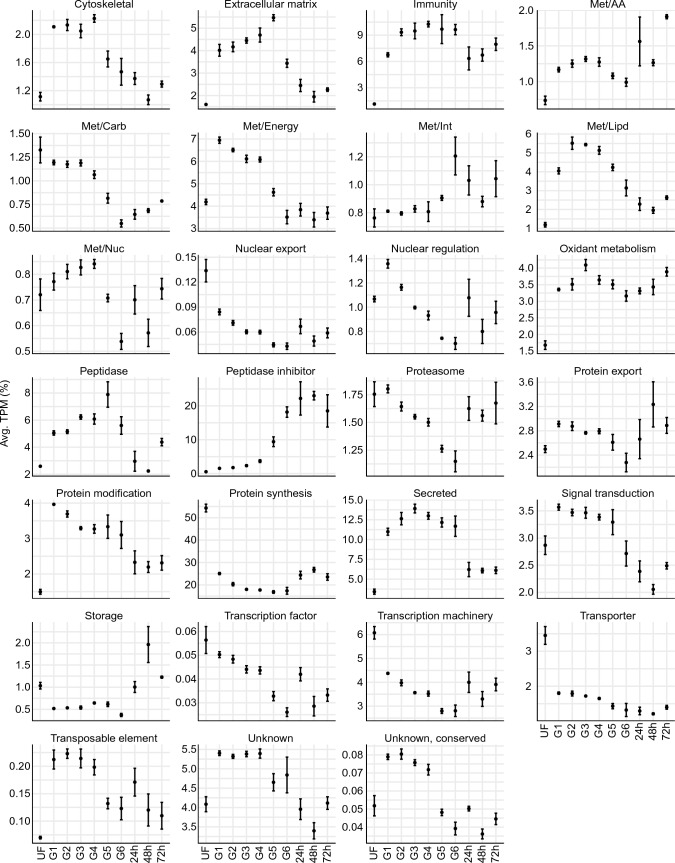
Relative quantification of the 27 functional classes over the different feeding stages of *I. scapularis* midgut. The average TPM (%) of each class was plotted against each biological group. The error bars represent the standard deviation of the mean. Plots were generated using the ggplot2 (3.4.2) package for R^23^.

The final set of 10,080 CDS was annotated and classified into 27 functional classes. Analysis of the relative quantification of each functional class at a given feeding stage (Fig. 2) illustrates how different metabolic processes are temporally organized during tick feeding, providing direct insights into tick midgut physiology. The "protein synthesis" functional group was found to be the first or second most abundant class in all feeding stages, indicating the highly active state of the tick midgut. Starting from group G6 and onwards, the "peptidase inhibitors" class was the most abundant. Notably, G6 represents ticks in the rapid feeding phase, during which a large volume of blood is rapidly consumed. It is highly likely that several of the upregulated inhibitors are responsible for blocking active host peptidases involved in pathways that could be detrimental to tick fitness (e.g. blood clotting)^27,28^ and/or are related to the regulation of tick digestive peptidases^29–31^. As expected, the "peptidase" class, which includes transcripts encoding putative enzymes involved in hemoglobin digestion, increased as feeding progressed, reaching its peak during the engorgement period. This transcriptional profile aligns with the overall hemoglobinolytic activity observed in midgut extracts of *I. ricinus* ticks, where the highest activity was observed in engorged ticks^8^. Additionally, our classification strategy includes the "unknown, conserved" and "unknown" classes, encompassing transcripts that were not classified within the other groups. Transcripts in the "unknown, conserved" class exhibit a high degree of similarity to other previously deposited sequences of currently unknown function, while transcripts in the "unknown" class show no or low similarities with previously deposited sequences, representing potential unique tick sequences.

In addition to the functional clustering of putative CDS, we also conducted unsupervised clustering of transcripts based on their TPM values across the different feeding stages. This analysis resulted in the formation of 24 clusters (Supplementary Fig. 2, indicated by column N in Supplementary File 1), enabling the identification of sequences with similar transcriptional patterns and stage-specific transcripts. This could be particularly relevant in the context of vector-borne diseases, as their transmission typically occurs within 24–48 h of tick attachment^32^. Identification of highly expressed CDS in the unfed or early feeding groups could offer a cost-efficient strategy for selecting potential antigens for further studies.

**Figure 3:**
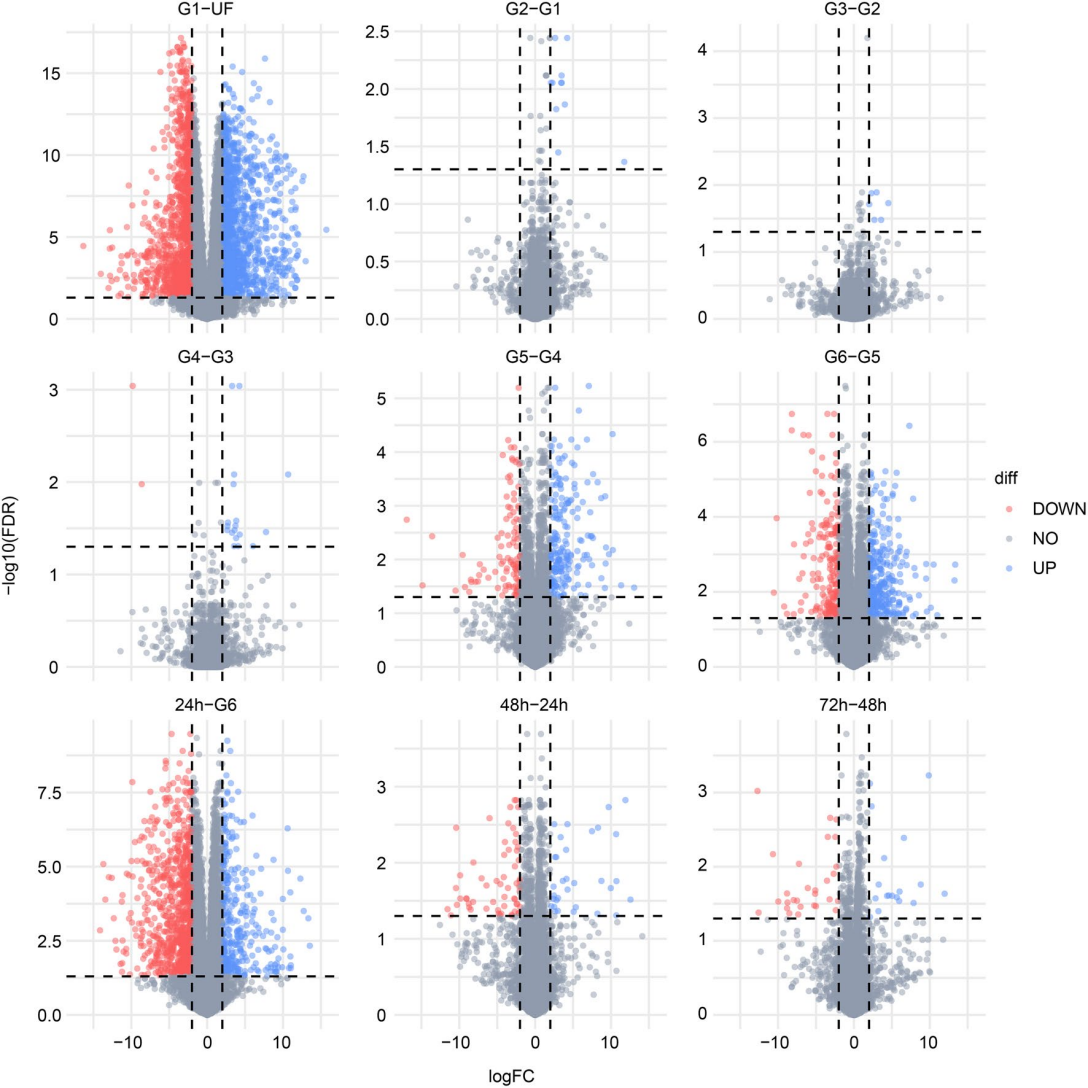
Volcano plots of the differentially expressed transcripts found between the pairwise comparison of different biological conditions. Statistical difference was considered when a transcript presented LogFC ≥  ± 2 (vertical dotted lines) and FDR ≤ 0.05 (horizontal dotted lines). The number inside the plots indicated the number of transcripts upregulated (blue) or down-regulated (red). Transcripts that were not considered differentially expressed are shown as gray dots. Plots were generated using the ggplot2 package for R^23^.

To gain a deeper understanding of the transcriptional changes induced by blood feeding, we performed pairwise differential expression analysis at each feeding stage compared to the preceding condition (Fig. 3). As expected, the initial contact of the tick midgut with host blood (G1—unfed) triggered the most significant transcriptional changes in the tissue, with a total of 2,545 modulated transcripts. Interestingly, most of these induced changes were maintained throughout the slow-feeding phase, with only a few differentially expressed transcripts between G2/G1, G3/G2, and G4/G3 pairwise comparisons. Additional transcriptional changes were observed between G5/G4 (300 transcripts) and G6/G5 (483 transcripts), representing the transition to the rapid-feeding phase. The "big sip" occurs in the final 24 h of feeding, during which the tick ingests a substantial amount of blood before detaching from the host. In our dataset, this stage is represented by the 24-hpd/G6 comparison, which revealed a total of 1,194 modulated transcripts. Finally, the comparisons between 48-hpd/24-hpd and 72-hpd/48-hpd showed 91 and 46 modulated transcripts, respectively, confirming the overall consistency of the transcriptional profile of the *I. scapularis* midgut in the early days post-detachment.

Collectively, the data presented in this study have allowed us to identify four main transcriptional profiles associated with the unfed, slow-feeding (G1-G4), rapid-feeding (G5-G6), and early post-detachment (24–72 hpd) phases of tick feeding. It is plausible that additional changes occur in the midgut of female ticks during a "late post-detachment" period (> 72 hpd), and further expansion of our experimental design will be necessary to investigate these changes. Furthermore, based on the average weight of the G5 group (44.5 mg) and the number of differentially expressed transcripts between G4/G5 and G6/G5, we can infer that the G5 group represents the transitional phase between slow-feeding and rapid-feeding.

In addition to providing a fundamental understanding of tick midgut physiology, the data presented in this study have implications for the identification of potential targets for immunological tick control. Currently, the only commercially available anti-tick vaccine is based on the midgut protein Bm86^11,12^, which serves as a proof of concept for the use of midgut proteins in anti-tick control strategies. Similarly, the LYMErix vaccine developed by SmithKline Beecham in the 1990s involved immunizing humans against Borrelia-derived OspA. This resulted in the production of circulating antibodies that, when ingested by ticks during a blood meal, bound and neutralized viable spirochetes in the tick midgut, thereby impeding their migration to the salivary glands and effectively preventing infection^33^. Therefore, the identification of midgut proteins that play a crucial role in spirochete migration from the midgut to the tick salivary glands may offer new opportunities for the development of tools to block pathogen transmission.

In the following subsections, we will discuss the main tick feeding stages, focusing on the differentially expressed transcripts and their functional classes.

### The midgut of unfed adult females

From a morphological standpoint, the midgut of unfed female adults of Ixodidae ticks can be described as small tube-like extensions with a barely visible lumen^34^. At this stage, the midgut mainly consists of a monolayer of undifferentiated reserved cells and degenerating digestive cells (dDC) that were preserved from the previous nymphal stage^35,36^. Resting digestive cells may also be observed, which are characterized by their small size, irregularly shaped nuclei, and smaller cytoplasmic vesicles compared to the dDC^37^.

Transcriptionally, the midgut of unfed adult *I. scapularis* females exhibited the most distinct profile when compared to the other groups (Fig. 1c and d). While the majority of the 10,080 transcripts had low TPM values, a group of transcripts was found almost exclusively in this stage (Fig. 1C), and they can be systematically identified through unsupervised clustering (Supplementary Fig. 1). Cluster 2 comprised 1110 transcripts (1065 CDS and 45 ncRNA) that were predominantly present in the unfed group. Cluster 3 (873 CDS and 19 ncRNA) displayed high TPM values in the unfed group and gradually decreased as feeding progressed, while transcripts in cluster 8 (385 CDS and 5 ncRNA) exhibited high abundance in the unfed group and moderate levels in the post-detachment feeding stages. Functional classification of the CDS within each cluster revealed that clusters 2 and 8 mainly consisted of transcripts related to "protein synthesis" (59% and 82%, respectively) and "transcription machinery" (7.4% and 3.1%, respectively), while cluster 3 was enriched in the "peptidase" (16.7%) and "metabolism of energy—Met/Energy" (14.4%) classes (Supplementary Fig. 2). It is likely that degenerating and resting digestive cells carried over from the nymphal stages are the main contributors to cluster 3.

The overall high abundance of transcripts related to protein synthesis suggests that the midgut of unfed ticks exhibits a "preparatory" transcriptional profile, priming the tissue for subsequent feeding stages when numerous proteins will be translated.

### The slow feeding phase (G1–G4)

As the feeding progresses, the tick midgut undergoes profound morphological and transcriptional changes. During this stage, the midgut lumen gradually expands as the host blood is taken up. The epithelium grows, and the undifferentiated reserved cells begin to differentiate into initial digestive and secretory cells^34,38^. Hemoglobin is continuously internalized into the digestive cells, which increase significantly in size compared to those of unfed ticks^37^. The digestive cells exhibit distinct uptake mechanisms for hemoglobin and albumin, the major components of the blood meal. Albumin is incorporated into small vesicles (1–2 µm) through non-specific endocytosis, while hemoglobin appears to be specifically recognized by a yet-to-be-discovered cell surface receptor and integrated into larger endosomes (3–12 µm)^6^. This stage also marks the initiation of hemoglobin digestion and the formation of hemosomes^8^.

The slow feeding phase extends over multiple days, and thus we collected ticks with different average weights (G1 = 7.4 mg, G2 = 15.9 mg, G3 = 24.6 mg, and G4 = 44.5 mg) to capture potential transcriptional changes within this phase, in addition to the changes triggered by the initial contact of the tick midgut with host blood. As expected, the differential expression analysis between G1 and the unfed group showed the highest number of transcriptional changes (Fig. 3). However, subsequent comparisons between the groups within the slow-feeding phase revealed minimal differences. Therefore, we can summarize the transcriptional profile of the slow-feeding phase based on the G1/unfed comparison. Functional classification of the 2,460 differentially expressed CDS (Table 1) revealed an overall up-regulation of transcripts related to "immunity" (15.5-fold), "peptidase" (6.7-fold), and "protein modification" (5.75-fold) classes, while the most down-regulated classes were "nuclear export" (0.22-fold), "transporters" (0.22-fold), and "transcription machinery" (0.26-fold). It is important to note that in addition to the 2,460 CDS, we also observed the differential expression of 89 sequences annotated as ncRNA (Table 1). The function of ncRNA in tick physiology is a relatively new area of research and remains largely unexplored^39,40^. In other hematophagous vectors, such as mosquitoes, several putative ncRNAs have been reported and are believed to play important regulatory roles in epigenetic, transcriptional, and post-transcriptional gene processes^41,42^. Similarly, the modulated ncRNAs identified here are likely to have important regulatory roles in tick midgut physiology.

**Table 1:** Functional classification of the differentially expressed transcripts between the G1 and unfed group.

Class	No. of transcripts		TPM UF	TPM G1	G1/UF
	Up	Down			
Immunity	31	23	3295.72	51,239.32	15.55
Peptidase	60	36	2726.04	18,262.68	6.70
Protein modification	62	20	3591.39	15,357.39	5.75
Transposable element	16	16	277.25	1427.62	5.15
Secreted	77	95	22,209.05	101,958.72	4.59
Cytoskeletal	58	43	2091.56	6543.15	3.13
Extracellular matrix	74	18	12,554.16	35,003.23	2.79
Met/AA	23	17	1356.21	2858.97	2.11
Oxidant metabolism	49	95	10,721.39	21,430.74	2.00
ncRNA	47	42	3434.83	4997.76	1.46
Signal transduction	104	88	7,115.38	8869.21	1.25
Met/Int	26	18	2604.54	2959.63	1.14
Unknown	172	164	16,235.30	16,326.18	1.01
Protein export	49	55	3378.88	3288.79	0.97
Met/Energy	49	26	9550.05	8018.08	0.84
Unknown, conserved	3	3	111.89	91.34	0.82
Nuclear regulation	47	43	4456.84	2891.95	0.65
Met/Lipd	73	49	227,002.52	134,534.71	0.59
Met/Carb	33	25	7745.66	3961.86	0.51
Met/Nuc	20	19	223,861.92	113,135.74	0.51
Peptidase inhibitor	23	14	208,803.6	100,071.8	0.44
Storage	8	3	9298.47	3901.47	0.42
Proteasome	29	41	5533.98	2319.70	0.42
Transcription factor	1	5	78.59	28.39	0.36
Protein synthesis	25	67	200,634.39	70,107.78	0.35
Transcription machinery	59	154	29,075.67	7475.10	0.26
Transporter	60	79	24,415.65	5449.79	0.22
Nuclear export	4	5	861.79	188.69	0.22

Further exploration of the immune-related CDS revealed that the most upregulated transcripts (LogFC = 4.8 − 8.3, Supplementary File 2) contained a ML-domain (PFAM 2221), which has been implicated in pathogen recognition by binding to specific surface lipids^43^. Several tick ML-domain containing sequences have been reported^44,45^, including one that is upregulated during tick feeding in *B. burgdorferi*-infected guinea pigs^46^. Although experimental evidence of their involvement in pathogen recognition is still lacking, it is likely that at least some of these sequences possess this activity. Thus, the initial contact with host blood appears to create a "surveillance environment" in the tick midgut, inducing CDS that are potentially involved in the identification of pathogens that may be acquired during the blood meal.

In addition to the ML-domain-containing proteins, four transcripts encoding putative defensins were also found to be upregulated in the G1 group (LogFC 2.0 − 2.7, Supplementary File 2). Several defensin-like proteins have been characterized in different tick species, and they have shown to be modulated during feeding and in response to bacterial challenge^47,48^, highlighting their contribution to pathogen control in the midgut. Accumulation of blood in the midgut lumen also creates a favorable environment for bacterial growth and is associated with the proliferation of bacterial communities in blood-feeding arthropods^49,50^. However, in *I. ricinus*, a reduction in 16S rDNA was observed as feeding progresses, suggesting that tick immunity might contribute to this reduction^51^. It is possible that the induced ML-domain containing sequences and defensins can also recognize the tick bacterial community and contribute to its control.

The second most upregulated functional group between the G1 and unfed groups was the "peptidases" (Table 1), which includes multiple enzymes involved in hemoglobin digestion. In hematophagous arthropods, blood digestion usually occurs in the midgut lumen and is primarily carried out by serine peptidases, with the meal being surrounded by the semipermeable peritrophic matrix/membrane^52^. However, in ticks, digestion occurs intracellularly, and hemoglobin degradation is orchestrated by an array of aspartic, cysteine peptidases, and carboxy peptidases^7,53^. As expected, we observed several CDS encoding peptidases of these classes throughout the slow-feeding phase (Supplementary Fig. 3). Interestingly, we observed moderate levels of cysteine peptidase transcripts in unfed ticks. However, considering that the midgut extract of unfed ticks displays low hemoglobinolytic activity , it is likely that these transcripts are only translated once blood intake begins or that the translated proteins are maintained as zymogens. Additionally, we observed a steady increase in CDS encoding putative serine peptidases belonging to the MEROPS serine peptidases family S01 during the slow-feeding phase, reaching their peak during engorgement (Supplementary Fig. 3). Similar findings have also been reported in the *I. ricinus* midgut transcriptome, where multiple serine peptidases were found at later feeding stages^14^. These enzymes could potentially play a role in the digestion of proteins other than albumin and hemoglobin in the midgut lumen, similar to what has been described in other hematophagous arthropods. However, at present, there is no experimental evidence supporting this hypothesis.

### Transition between the slow- to the rapid-feeding phase (G4–G5)

A definitive distinction between the end of the slow-feeding phase and the beginning of the fast-feeding phase poses a challenge due to their close temporal proximity. In our analysis, based on the average tick weight of G5 (44.5 mg) and the substantial number of differentially expressed transcripts between G5/G4 and G6/G5 (Fig. 3), we consider G5 as a reasonable representation of the transition between the slow- and rapid-feeding stages. Morphologically, the midgut of ticks at this stage appears larger, with digestive cells containing residual bodies, large endosomes, and lipid inclusions. Some of the digestive cells can be observed detached from the midgut epithelium, "floating" in the lumen^8^.

Differential expression analysis between G5/G4 revealed the modulation of 300 transcripts (202 upregulated and 98 down-regulated). Among the 25 functional classes found to be modulated in this comparison, 22 were upregulated (TPM_G5_/TPM_G4_ > 1, Table 2), indicating an overall high transcriptional profile in the tick midgut. The classes with the highest ratios between G5 and G4 were "ncRNA" (15.6-fold), "metabolism of nucleotide—Met/Nuc" (12.1-fold), and "metabolism of carbohydrates—Met/Carb" (7.4-fold). However, these ratios should be interpreted cautiously as transcripts within these classes had generally low TPM values in both G4 and G5 groups (Table 2), providing a misleading interpretation of the major changes in this feeding stage. The only exception was XR_005718073.1 (TPM_G4_ = 38.8, TPM_G5_ = 1,303), classified as an ncRNA, suggesting its potential role in tick midgut physiology. Other functional classes that exhibited increased expression in the G5 group and had high TPM values (> 10,000) were "secreted" (~ sevenfold), "peptidase inhibitor" (4.26-fold), and "peptidase" (3.6-fold) groups.

**Table 2:** Functional classification of the differentially expressed transcripts between the G5 and G4 groups.

Class	No. of transcripts		TPM G4	TPM G5	G5/G4
	Up	Down			
ncRNA	3	1	90.60	1410.13	15.56
Met/Nuc	1	–	6.97	84.27	12.10
Met/Carb	3	1	10.85	80.05	7.38
Secreted	19	12	1593.48	11,130.82	6.99
Protein export	6	2	10.31	71.18	6.90
Met/Int	12	–	103.98	692.78	6.66
Protein modification	8	1	1377.42	7933.37	5.76
Met/Energy	3	1	127.19	732.00	5.76
Extracellular matrix	7	10	1504.42	8005.11	5.32
Met/AA	6	–	175.21	863.27	4.93
Peptidase inhibitor	11	1	14,664.84	62,417.02	4.26
Proteasome	2	–	2.10	8.03	3.82
Peptidase	20	3	6485.64	23,582.44	3.64
Transporter	15	9	88.88	317.97	3.58
Immunity	3	2	66.00	227.18	3.44
Protein synthesis	1	1	39.59	130.08	3.29
Unknown	25	12	319.14	961.16	3.01
Transposable element	3	1	4.67	12.03	2.58
Cytoskeletal	6	3	43.04	109.03	2.53
Oxidant metabolism	14	12	312.67	603.28	1.93
Transcription machinery	7	4	92.91	153.06	1.65
Met/Lipd	16	5	841.99	1200.57	1.43
Signal transduction	10	13	185.16	86.68	0.47
Unknown, conserved	–	2	19.66	2.71	0.14
Storage	1	2	263.78	2.42	0.01

The "secreted" class comprises CDS with putative signal peptides, commonly found in the salivary glands and saliva of ticks^54^ and other blood-feeding vectors^55–59^. In the current analysis, the most abundant (TPM_G5_ > 3,500) and variable (LogFC > 3) transcripts within the "secreted" class were XP_029844885.2 and XP_029844870.2, which shared approximately 60% similarity in their primary structure (Supplementary Fig. 4A). Additionally, both transcripts were predominantly found in the G5 and G6 groups (Supplementary Fig. 4A), suggesting a potential role during tick engorgement. Currently, they are functionally annotated as "unknown, conserved" since they do not contain any known conserved domain, and BLAST searches against several reference databases only retrieved putative CDS from other ticks lacking functional characterization (Supplementary File 1). Together, they represent highly abundant, stage-specific, novel, and exclusive putative sequences from ticks.

Overall, the "peptidase inhibitor" class exhibited a highly consistent transcriptional profile across different feeding stages (Fig. 2). During the unfed and slow-feeding phases, they were present in low abundance and exponentially increased during the engorgement phase, reaching a plateau after detachment. In the G5/G4 comparison, the most abundant and modulated peptidase inhibitors were transcripts containing a Kunitz BPTI domain (Supplementary File 1). Currently, Kunitz-type inhibitors isolated from tick midguts have been shown to inhibit different host serine peptidases involved in blood clotting. Additionally, knockdown of such inhibitors resulted in impaired tick feeding fitness by prolonging or completely interrupting blood meal acquisition^27,28,60,61^. It has also been proposed that Kunitz-type inhibitors can target endogenous serine peptidases present in the tick midgut, regulating their proteolytic activity towards the blood meal^62^. A noteworthy feature is the temporal transcriptional regulation of these inhibitors. Their exponential increase during the end of the slow-feeding phase potentially leads to the accumulation of multiple inhibitors in the tick lumen just before or during the rapid engorgement period, when the tick ingests a large volume of blood and increases the host serine peptidases that need to be regulated. This specific transcriptional pattern indicates the presence of a sophisticated regulatory system in the tick midgut that is still largely unknown.

Interestingly, during the transition to the rapid-feeding phase, the most abundant peptidases in the tick midgut were trypsin-like serine peptidases (Supplementary Fig. 3). The role of serine peptidases in tick midgut physiology is currently elusive. In *Haemaphysalis longicornis*, two serine peptidases were induced by blood feeding and detected in the midgut lumen. Additional knockdown experiments resulted in reduced red blood cell hemolysis in the tick midgut^63,64^, suggesting that serine peptidases may be involved in the degradation of host red blood cells and the release of hemoglobin. Recently, it was demonstrated that midgut extracts from unfed and partially fed *I. scapularis* displayed negligible trypsin-like activity, while this activity peaked in ticks 1 day post-detachment^65^. The authors also conducted knockdown experiments of selected serine peptidases, resulting in decreased tick survival, and midgut extracts from silenced ticks exhibited reduced activity towards hemoglobin, indicating the potential contribution of serine peptidases in later feeding stages. The asynchronous peaks between transcript abundance and protein activity of serine peptidases could be attributed to either delayed translation or the presence of proteins being translated and maintained as zymogens in the tick midgut. These zymogens are later activated by an unknown signaling pathway.

### Rapid-feeding phase (G5–G6)

Also referred to as the 'big sip,' the rapid-feeding phase occurs during the last 12 to 24 h of feeding. During this period, mated female ticks consume a large volume of blood, resulting in rapid and significant expansion of their bodies. In some cases, fully fed females can increase their body weight by approximately 100 times compared to their unfed state^34^. At this stage, the midgut lumen becomes completely filled with blood, and the digestive cells stretch and contain multiple cytoplasmic vesicles^37^.

In the overall analysis of differential expression between the G6 and G5 groups, a similar pattern to the G5/G4 comparison was observed. The most abundant and modulated functional classes in G6/G5 were "peptidase inhibitor," "secreted," "peptidase," and "ncRNA" (Table 3). Out of the 26 functional groups, 19 were found to be upregulated (TPM_G6_ / TPM_G5_ > 1, Table 3), indicating a slight reduction in the overall transcriptional activity of the tick gut compared to the previous stage (G5/G4: 22 out of 25 classes were upregulated). In G5, the predominant peptidase inhibitors belonged to the Kunitz subfamily. However, in G6, two transcripts accounted for 98.8% of all quantified inhibitors (Supplementary file 2). The transcripts XP_040077223.1 (TPM_G6_ = 12,771.2) and XP_040077225.2 (TPM_G6_ = 6,851.1) were among the most abundant transcripts in the midgut of engorged ticks and are currently annotated as trypsin-inhibitor-like (TIL, CDD: CD19941). TIL-domain containing proteins have been isolated from tick eggs and hemolymph and, in addition to their serine peptidase inhibitory activity, have displayed antimicrobial properties^66,67^. This highlights their multiple roles in tick physiology as regulators of host and/or endogenous serine peptidases, in addition to their potential to control pathogen proliferation. Both transcripts contain a single TIL domain and exhibit a methionine at the putative P1 position (Supplementary Fig. 4B), similar to the *Apis mellifera* chymotrypsin inhibitor (AMCI)^68^. Therefore, these inhibitors may target host-derived chymotrypsin-like peptidases present in the ingested blood meal, such as cathepsin G and chymase.

**Table 3:** Functional classification of the differentially expressed transcripts between the G6 and G5 groups.

Class	No. of transcripts		TPM G5	TPM G6	G6/G5
	Up	Down			
ncRNA	16	6	668.65	6541.63	9.78
Peptidase inhibitor	6	2	2173.80	19,858.68	9.14
Proteasome	5	1	6.84	60.28	8.82
Transcription machinery	12	5	109.05	960.73	8.81
Met/Int	24	4	468.32	3937.52	8.41
Protein export	8	3	199.61	1261.09	6.32
Cytoskeletal	8	2	28.03	164.58	5.87
Transposable element	13	2	41.22	195.33	4.74
Met/AA	6	1	37.32	165.48	4.43
Nuclear regulation	1	–	1.34	5.20	3.87
Peptidase	15	11	3311.40	10,818.15	3.27
Unknown	43	25	2181.97	6566.88	3.01
Oxidant metabolism	29	18	2066.31	5142.87	2.49
Immunity	7	9	3641.04	8753.72	2.40
Transporter	17	17	585.06	1212.85	2.07
Signal transduction	17	4	154.10	233.89	1.52
Secreted	38	13	12,310.35	18,552.07	1.51
Protein synthesis	4	3	53.51	72.77	1.36
Storage	5	2	202.09	213.72	1.06
Met/Lipd	10	14	2529.97	1785.44	0.71
Met/Nuc	2	2	94.39	48.26	0.51
Met/Carb	3	8	977.55	256.89	0.26
Protein modification	1	9	3507.95	767.20	0.22
Met/Energy	1	3	546.68	106.65	0.20
Transcription factor	–	1	0.98	0.17	0.17
Extracellular matrix	3	24	6282.12	501.11	0.08

Similar to the G5 group, engorged ticks (G6) exhibited several upregulated trypsin-like serine peptidases (Supplementary File 2). However, these transcripts differed from those observed in the G5/G4 comparison, suggesting the accumulation of multiple serine peptidases in the tick midgut toward the end of the feeding period^65^. Furthermore, the presence of "stage-specific" peptidases within the same family resembles the phenomenon of "early" and "late" trypsins described in mosquitoes^69^ or the cysteine peptidases from *Rhodnius prolixus*^70^. The transcriptional pattern of the "peptidase inhibitors" class and the modulation of multiple trypsins in different feeding stages highlight the existence of a sophisticated regulatory mechanism governing blood meal digestion in ticks.

### Early detachment phase (24 h–G6)

Since it is well established that some tick-borne pathogens, such as *B. burgdorferi*, are typically transmitted within 48 to 72 h of tick attachment^32^, most studies on tick physiology have focused on the early stages of feeding, including attachment and slow-feeding phases. Consequently, our understanding of tick physiology remains incomplete. Tick detachment represents a significant event in the tick life cycle, and from a transcriptional perspective, this stage showed the second highest number of modulated transcripts (24 h/G6 comparison: 328 upregulated and 829 down-regulated).

During the transition from unfed to engorged ticks, the majority of functional classes were found to be upregulated (Tables 1, 2 and 3). However, upon detachment, we observed a drastic shift in the transcriptional activity of the *I. scapularis* midgut. Out of the 28 functional groups, 23 of them were down-regulated (TPM_24hpd_/TPM_G6_ < 1). Furthermore, the upregulated classes, namely "storage," "transcription factor," "proteasome," "metabolism of amino acids—Met/AA," and "nuclear regulation," exhibited low to moderate TPM values (Table 4), indicating an overall reduction in transcriptional activity at this stage. The upregulated transcripts in the "storage" class primarily encoded putative hemelipoproteins (Supplementary file 2) and were highly abundant at all time points post-detachment. Hemelipoproteins are large proteins (> 200 kDa) capable of binding heme, lipids, and carbohydrates. They are primarily synthesized by the tick fat body^71,72^, while the midgut serves as a secondary production site^73^. Besides their role in embryo development^73,74^, hemelipoproteins play a crucial role in heme metabolism in ticks. It has been demonstrated that essential enzymes involved in heme biosynthesis are absent in ticks^75^, rendering them reliant on exogenous sources of this prosthetic group^76^. Therefore, the accumulation of hemelipoproteins in the tick midgut after engorgement would aid in heme detoxification while providing the tick with this essential molecule^77^.

**Table 4:** Functional classification of the differentially expressed transcripts between the 24-hpd and G6 groups.

Class	No. of transcripts		TPM G6	TPM 24 h	24 h/G6
	Up	Down			
Storage	5	1	590.92	5467.09	9.25
Transcription factor	1	–	1.28	7.79	6.10
Proteasome	8	10	240.20	1357.26	5.65
Met/AA	7	9	1511.44	5417.31	3.58
Nuclear regulation	11	7	83.78	148.58	1.77
Transcription machinery	43	34	1254.25	1222.18	0.97
Oxidant metabolism	25	74	9202.11	7461.87	0.81
Protein synthesis	11	5	305.55	227.43	0.74
Immunity	13	22	13,044.87	9221.52	0.71
Met/Energy	6	10	278.40	161.04	0.58
ncRNA	7	29	8130.48	4226.21	0.52
Met/Nuc	3	7	404.88	204.38	0.50
Met/Carb	7	12	803.24	271.90	0.34
Met/Int	9	14	1996.51	664.23	0.33
Secreted	24	121	57,414.35	17,864.30	0.31
Cytoskeletal	9	27	2368.60	659.71	0.28
Transposable element	3	15	127.25	27.99	0.22
Peptidase inhibitor	6	23	31,699.07	6437.76	0.20
Transporter	22	74	3125.02	616.69	0.20
Unknown	44	87	17,566.34	3266.55	0.19
Signal transduction	22	57	6744.88	1075.42	0.16
Peptidase	16	52	26,552.38	4184.18	0.16
Met/Lipd	12	51	15,258.53	1921.12	0.13
Nuclear export	–	2	3.90	0.49	0.13
Unknown, conserved	–	3	12.95	1.56	0.12
Protein export	11	29	4512.35	443.59	0.10
Protein modification	6	14	12,892.02	1148.16	0.09
Extracellular matrix	3	69	12,937.22	440.88	0.03

Interestingly, when comparing the later time points after tick detachment, we observed a small number of differentially expressed transcripts. In the 48 hpd / 24 hpd comparison, a total of 91 transcripts (59 down and 32 up) exhibited modulation, while in the 72 hpd/48 hpd comparison, only 46 transcripts showed modulation (Fig. 3). Further examination of the differentially expressed transcripts (Supplementary File 2) revealed that most of them had low levels of TPM (< 1000), representing a minor fraction of the transcripts present in the tick midgut at this stage. The only exception was the transcript XP_029829874.2 (TPM_72hpd_ = 4978), which encodes a putative antimicrobial peptide similar to microplusin^78^. Notably, until 48 hpd, XP_029829874.2 displayed marginal levels of TPM (maximum average TPM from UF to 48 hpd was 718.8). This stage-specific increase in transcription is intriguing, particularly because it has been demonstrated that ovaries at 72 hpd, but not ovaries of partially and fully engorged *R. microplus* ticks, exhibit high mRNA levels of microplusin^79^. It is important to highlight that transcript quantification does not always correlate with the actual protein concentration in a given sample. Despite the apparent transcriptional consistency observed in the tick midgut from 24 to 72 hpd, proteomic studies will be essential for assessing the activities and proteins present in the tick midgut during the early days following detachment.

### A deeper insight into the regulation of digestive peptidases of *I. scapularis* adult females

It is well established that aspartic and cysteine peptidases are the primary digestive enzymes in ticks, and numerous proteins from various species have been isolated and characterized^80–83^. More recently, serine peptidases have also been identified in the tick midgut, and their potential involvement in hemoglobin (Hb) digestion is currently being investigated^63–65^. The current digestive model proposed for *I. ricinus*^53^ serves as a fundamental framework for understanding how ticks process Hb. Cathepsin D-like enzymes, supported by legumain and cathepsin L-like enzymes, are responsible for the initial cleavage of Hb. On the other hand, large Hb fragments are further processed primarily by cathepsin B-like peptidases, followed by cathepsin C-like enzymes, carboxy peptidases, and leucine aminopeptidases, resulting in the production of dipeptides and free amino acids.

**Figure 4:**
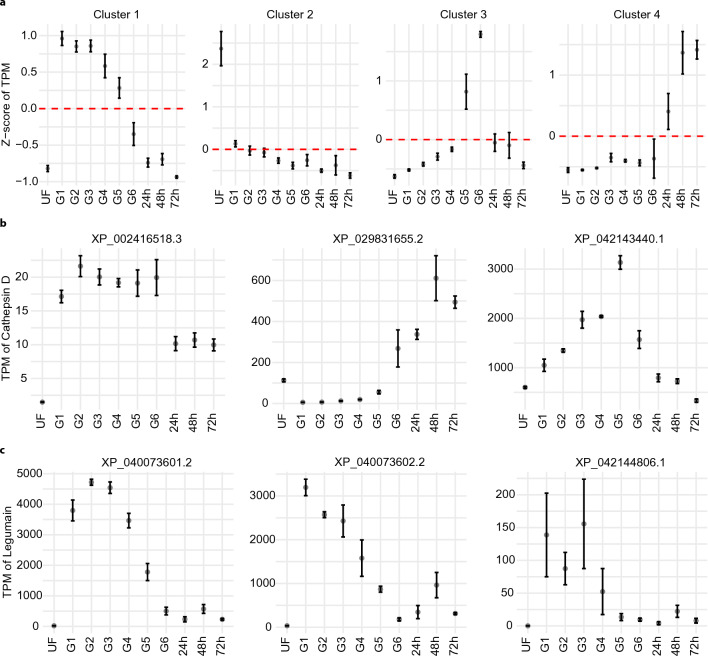
(**a**) Unsupervised clustering of transcripts that code putative digestive peptidases of *I. scapularis*. The dot represents the average Z-score of the TPM from the transcripts contained within the cluster, and the red dotted line marks the zero position in the y-axis. (**b**) Transcriptional profile of CDS coding for putative cathepsin D-like and (**c**) legumain-like peptidases. The dots represent the average TPM value found in each feeding stage. The error bars represent the standard deviation of the mean. Plots were generated using the ggplot2 package for R^23^.

It is also well known that peptidase activity must be strictly regulated to avoid detrimental consequences^84^. Common in vivo regulatory mechanisms include gene expression regulation, production of peptidases as zymogens, and the presence of endogenous inhibitors. In this study, we provide a comprehensive overview of the transcriptional patterns of several putative digestive enzyme transcripts at different feeding stages. The unsupervised clustering of these putative peptidases resulted in the formation of four main clusters, representing highly abundant peptidases in each feeding stage: Unfed, slow-feeding, rapid-feeding, and early post-detachment (Fig. 4a). Further exploration of each cluster revealed the presence of transcripts encoding peptidases from different families, indicating temporal regulation within peptidase families. One example is the cathepsin D-like peptidases. We identified three cathepsin D-like sequences, with two exhibiting medium to high TPM levels; however, their transcriptional profiles were almost complementary. XP_029831655.2 was virtually absent from the tick midgut until engorgement and reached its peak transcriptional level during the post-detachment phase, while XP_042143440.1 was moderately expressed in unfed ticks and showed a continuous increase during the slow-feeding phase until engorgement, followed by a sharp decrease upon detachment (Fig. 4b). Similar longitudinal regulation was observed for cathepsin B-like peptidases (Supplementary Fig. 5) and trypsin-like peptidases (Supplementary Fig. 6), but not for legumain-like peptidases, which were predominantly expressed during the slow-feeding phase (G1–G4, Fig. 4c). Further exploration of their UTR and genomic organization, coupled with the identification and characterization of tick transcriptional factors, will enhance our understanding of their stage-specific expression. Similarly, functional characterization of these peptidases will provide insights into substrate preferences and their potential roles during blood feeding.

The regulation of peptidase activity by endogenous inhibitors has also been demonstrated in several tick species, primarily for cysteine peptidases. These inhibitors are classified within the cystatin superfamily based on their primary features^86,87^, and multiple members have been identified in the tick midgut, associated with the regulation of hemoglobin degradation^29–31,71,85^. In the current dataset, we identified 14 coding sequences (CDS) encoding putative cystatins with varying transcriptional patterns (Supplementary Fig. 7). Overall, cystatins were moderately expressed during the slow-feeding phase and reached their peak expression during the G5 and G6 groups (engorgement). This pattern is somewhat contrary to the observed trend for cysteine peptidases (Supplementary Fig. 3), which showed a rapid decrease in expression during the engorgement phase. Essentially, this indicates the combination of two main regulatory systems (reduction of peptidase transcription and increase of inhibitors) to limit the proteolytic activity of cysteine peptidases in the tick midgut.

## Conclusion

Ticks have evolved a unique feeding strategy to acquire their blood meal, distinct from other blood-feeding arthropods, by remaining attached to their hosts for extended periods. Throughout the process of blood feeding, the midgut of adult female ticks undergoes significant morphological changes. In our study, we have demonstrated that these morphological changes are accompanied by equally dramatic transcriptional changes in the tick midgut. By collecting ticks at different weights corresponding to specific feeding stages (unfed, slow-feeding, rapid-feeding, and early post-detachment), we were able to characterize and assign specific transcriptional profiles to each stage. We believe that this comprehensive dataset, encompassing temporal and organ-specific information, will serve as a solid foundation for researchers interested in investigating tick midgut physiology. Furthermore, this dataset holds potential for identifying stage-specific transcripts and potential targets for the development of tick control strategies.

## Supplementary Information

Supplementary Figures.

Supplementary Information.

## Data Availability

The transcriptome data was deposited to the National Center for Biotechnology Information (NCBI) under Bioproject PRJNA876943 and Biosample accession SAMN33025934—SAMN33025963. The raw reads were deposited to the Short Reads Archive of the NCBI under accessions SRR21429759—SRR21429788. To facilitate the exploration of this dataset, we developed a R shiny application that can be accessed online (http://stephenlu.shinyapps.io/IsMg). Additionally, all supplementary files can be downloaded as a single compressed (.zip) file from the link: https://proj-bip-prod-publicread.s3.amazonaws.com/transcriptome/IsMg_2023/IsGutSupFiles.zip.
