# Supplementary material for: A longitudinal transcriptomic analysis from unfed to post-engorgement midguts of adult female Ixodes scapularis
Source: Sci Rep. 2023 Jul 13;13:11360. doi: 10.1038/s41598-023-38207-5 (PMC10345007; doi:10.1038/s41598-023-38207-5)
Supplement: Supplementary file 1 — Supplementary Figures. [file 41598_2023_38207_MOESM1_ESM.pdf]

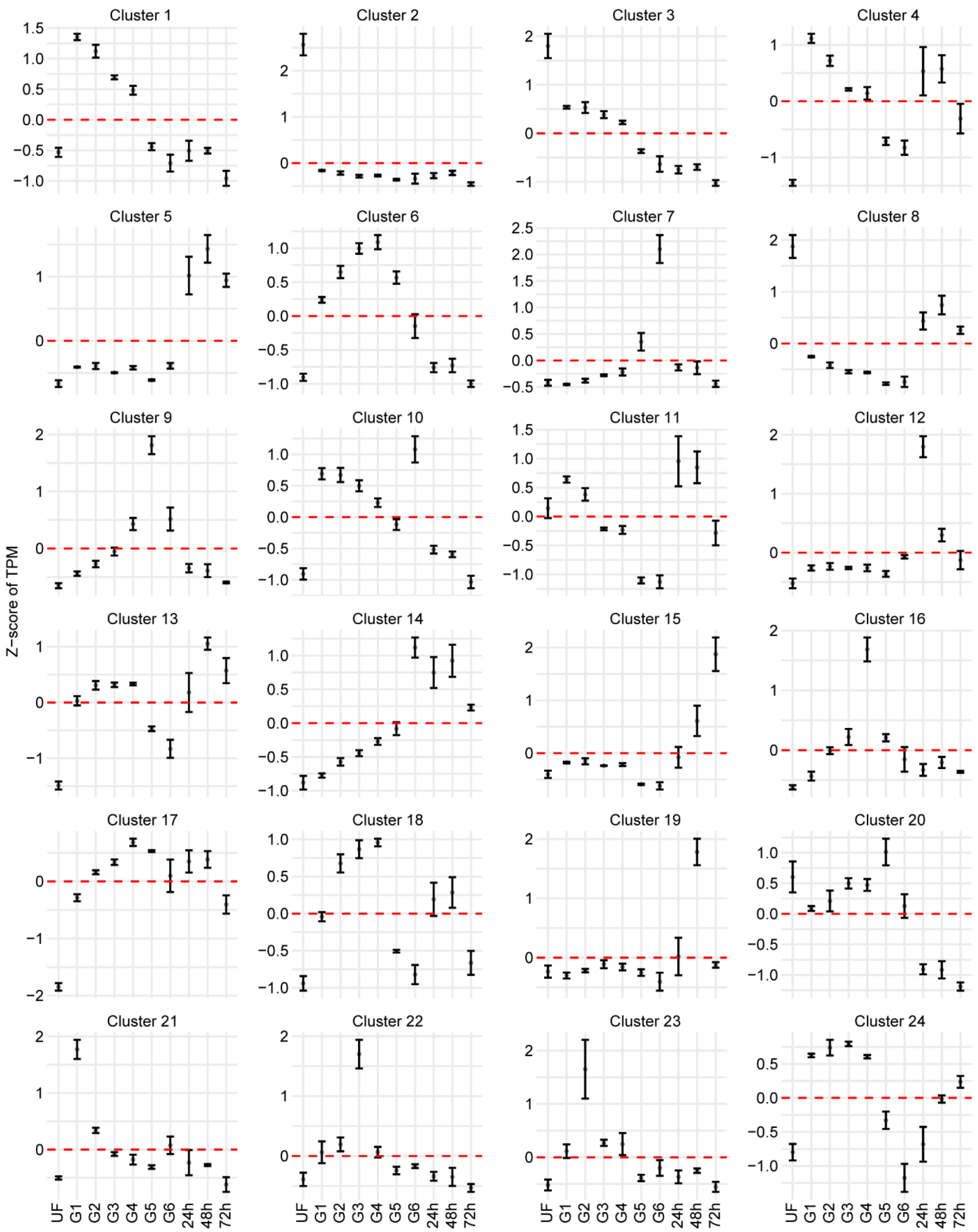

Supplementary Figure 1: Unsupervised clustering of transcripts that presented an average TPM ≥ 5 in at least one biological condition. The dots represent the average Z-

score of the TPM from the transcripts contained within the cluster and the error bars indicate the standard deviation of the mean. The red dotted line marks the zero position in the y-axis.

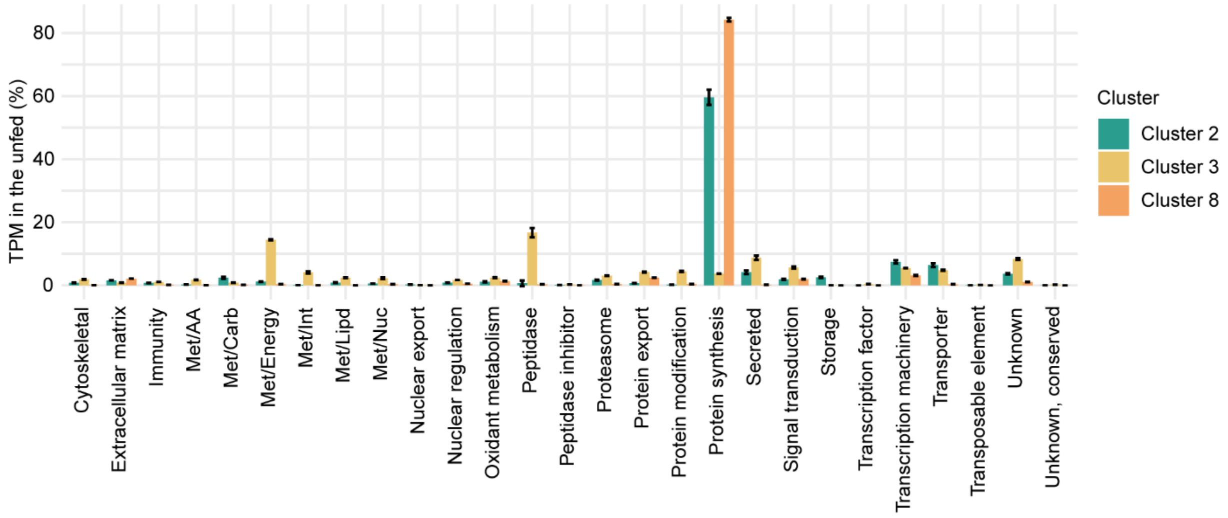

Supplementary Figure 2: Quantification of the functional classes within clusters highly abundant in the midgut of unfed ticks. Bars represent the average TPM of the transcripts classified within a functional class and the error bars represent the standard error of the mean.

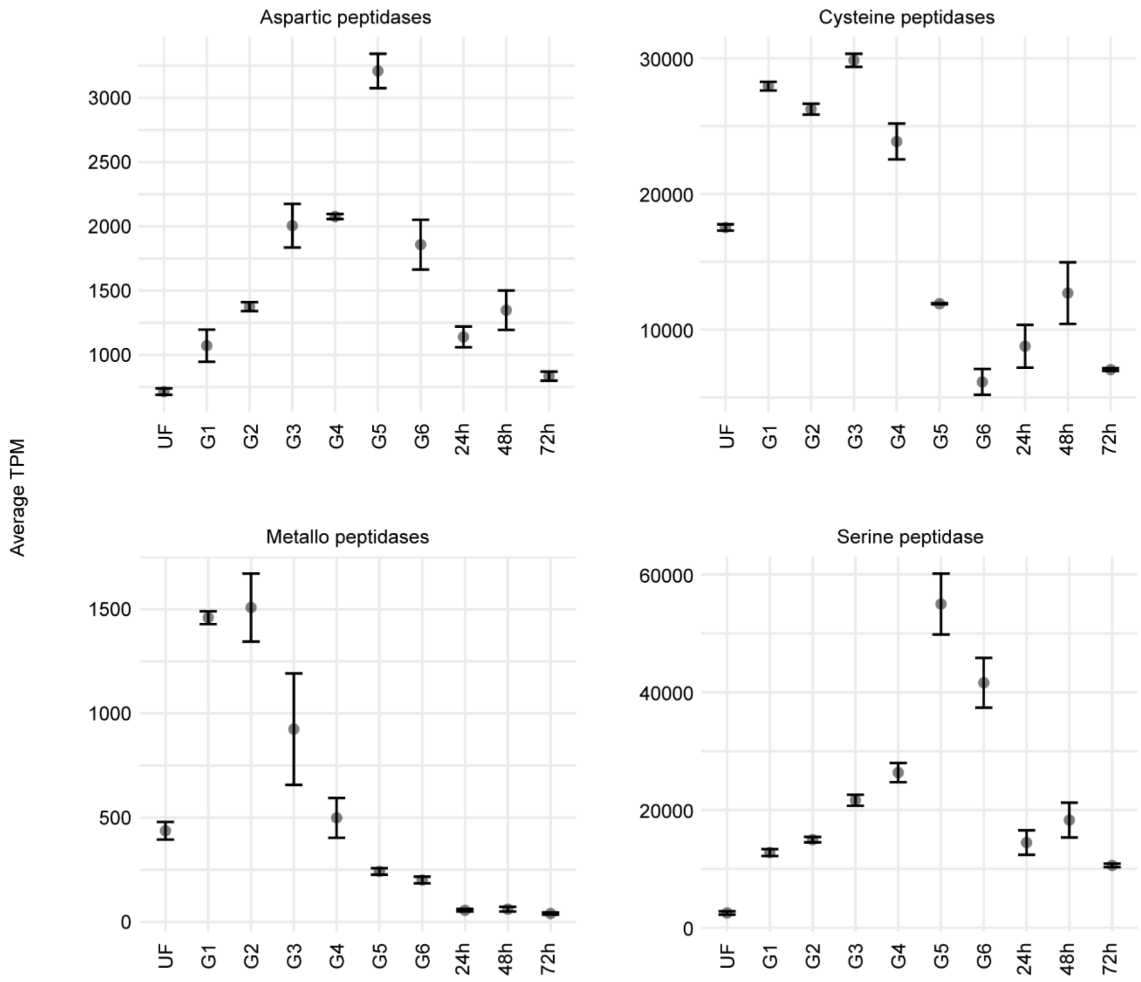

Supplementary Figure 3: Transcriptional profile of major peptidases families found in the midgut of *I. scapularis* adult females at different feeding stages. Dots represent the average TPM at each feeding stage and error bars represent the standard error of the mean.

a

```
XP_029844885.2 : IPTLDLTIEKEVEDAIRAEDPDTDSE------------------------ :  26
XP_029844870.2 : KSTVDRKIEQEPDESVTTDASDTDSVSDVTTAHEDNDLTTEASYGDSVSD :  50

XP_029844885.2 : --------------------SDPNLRKGEIYQEIGRKLKDEANAPHARGK :  56
XP_029844870.2 : ATTENWEEVVTTAASDKGGISDANIQKGEMFQEIGRQVKAIANHTETSPK : 100

XP_029844885.2 : KVSGFKRFWRAVKRAFKEACKAFVKTGKEAFIKEIEVEFEEEIGKVKKAF : 106
XP_029844870.2 : KVSKIKRFFRAVKKAFKEACKAFVKTGKEAFIREVNAEFEEEIGKVKKTF : 150

XP_029844885.2 : EMYSLDEKTTYQQLMLDFADTMELIGAKYIAKGRKQVA-------- : 144
XP_029844870.2 : GMYSLDEETTYQHMLLSFAKDMDLIGSIYIAKGRKQNTANEATRLQ : 196
```

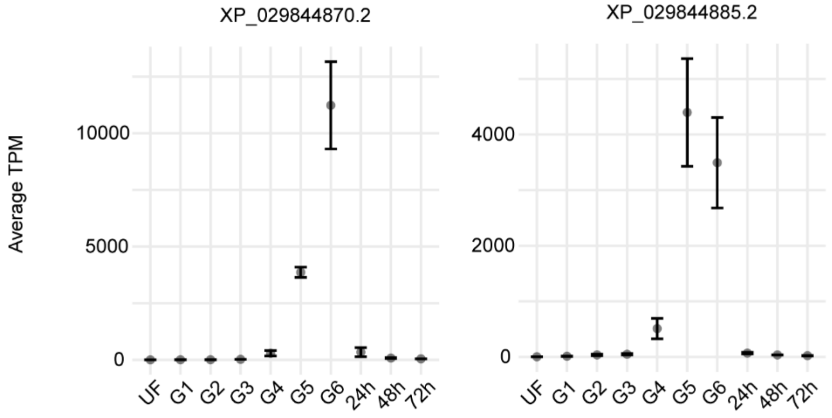

b

```
                                                       *
XP_040077223.1 : AADLPWVCGPREVFKECVSSSCAELKCGMEGMPEACTMDCASGCFCAPGF :  50
XP_040077225.2 : PADLPWVCGPREVFKECVSSSCAELKCGIEGMPEACTMDCASGCFCAPGF :  50
1CCV           : -----EECGPNEVFNTCG-SACAP-TCAQP-KTRICTMQCRIGCQCQEGF :  42

XP_040077223.1 : YRKGHRECVPWSECQIEPLKPMPKPXTNFKAVPDLSCXSELLLNKTXIVG : 100
XP_040077225.2 : YRRGHRECVPQSECQIEPLKPMPYA------------------------- :  75
1CCV           : LRNGEGACVLPENC------------------------------------ :  56

XP_040077223.1 : EAIFVFLCFIREFVRSEA : 118
XP_040077225.2 : ------------------ :   -
1CCV           : ------------------ :   -
```

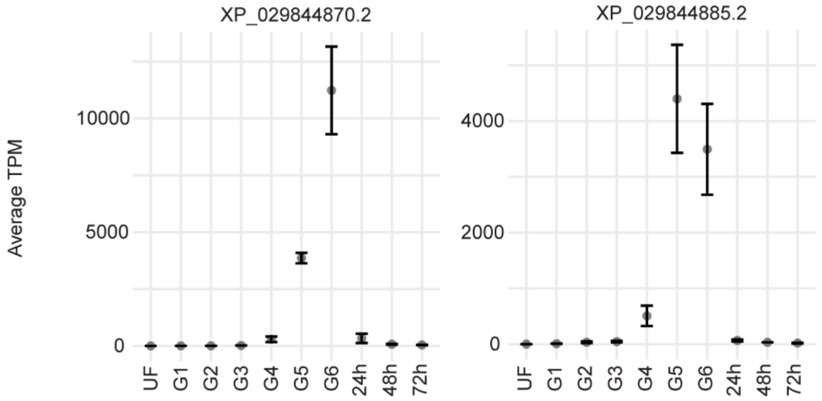

Supplementary Figure 4: (A): Alignment of XP_029844885.2 and XP_0298448870.2 amino acid sequences and their transcript quantification at the different feeding stages. **(B):** Alignment of the trypsin-inhibitor like (TIL) transcripts XP_040077223.1 and

XP_040077225.2 with the *Apis melifera* AMCI (PDB: 1CCV). Identical (black) and similar (gray) residues are highlighted. Dots represent the average TPM, and the error bars represent the standard error of the mean.

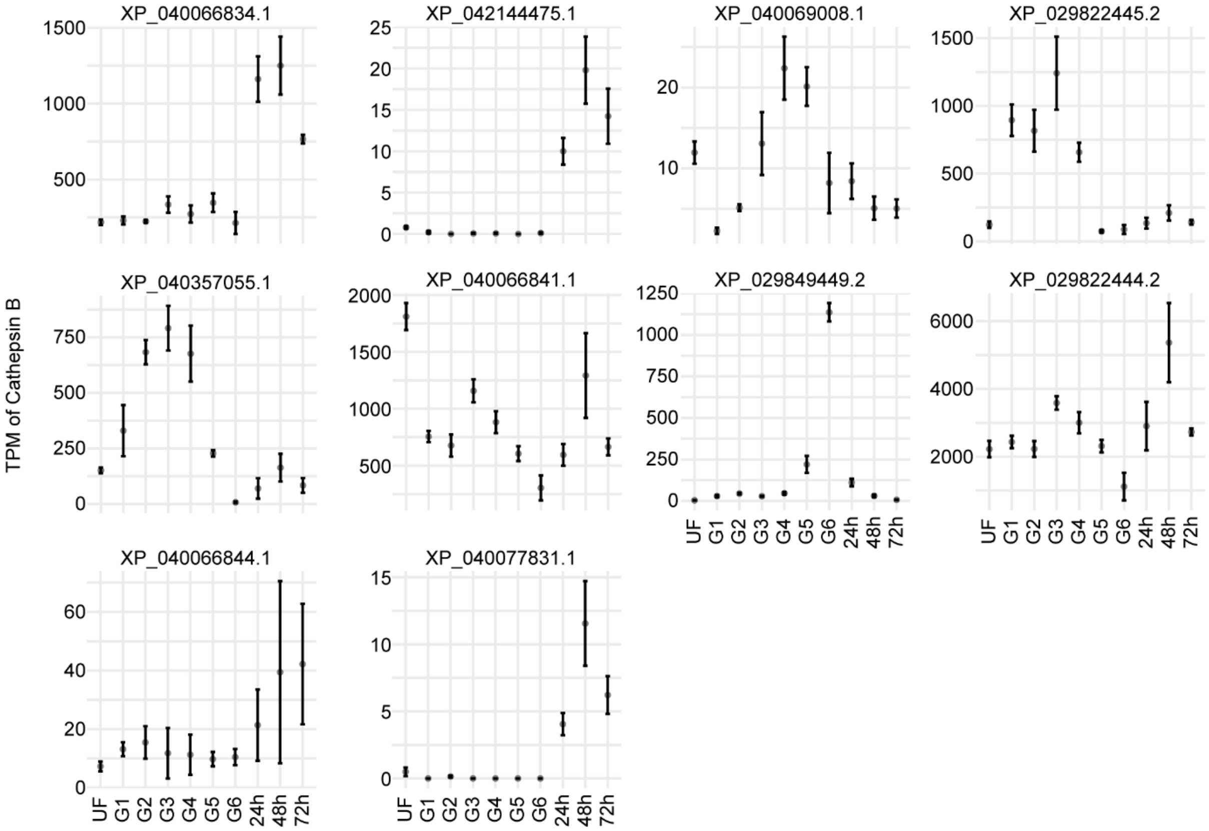

Supplementary Figure 5: Transcriptional profile of CDS coding for putative cathepsin B-like peptidases. The dots represent the average TPM value found in each feeding stage, and the error bars represent the standard deviation of the mean.

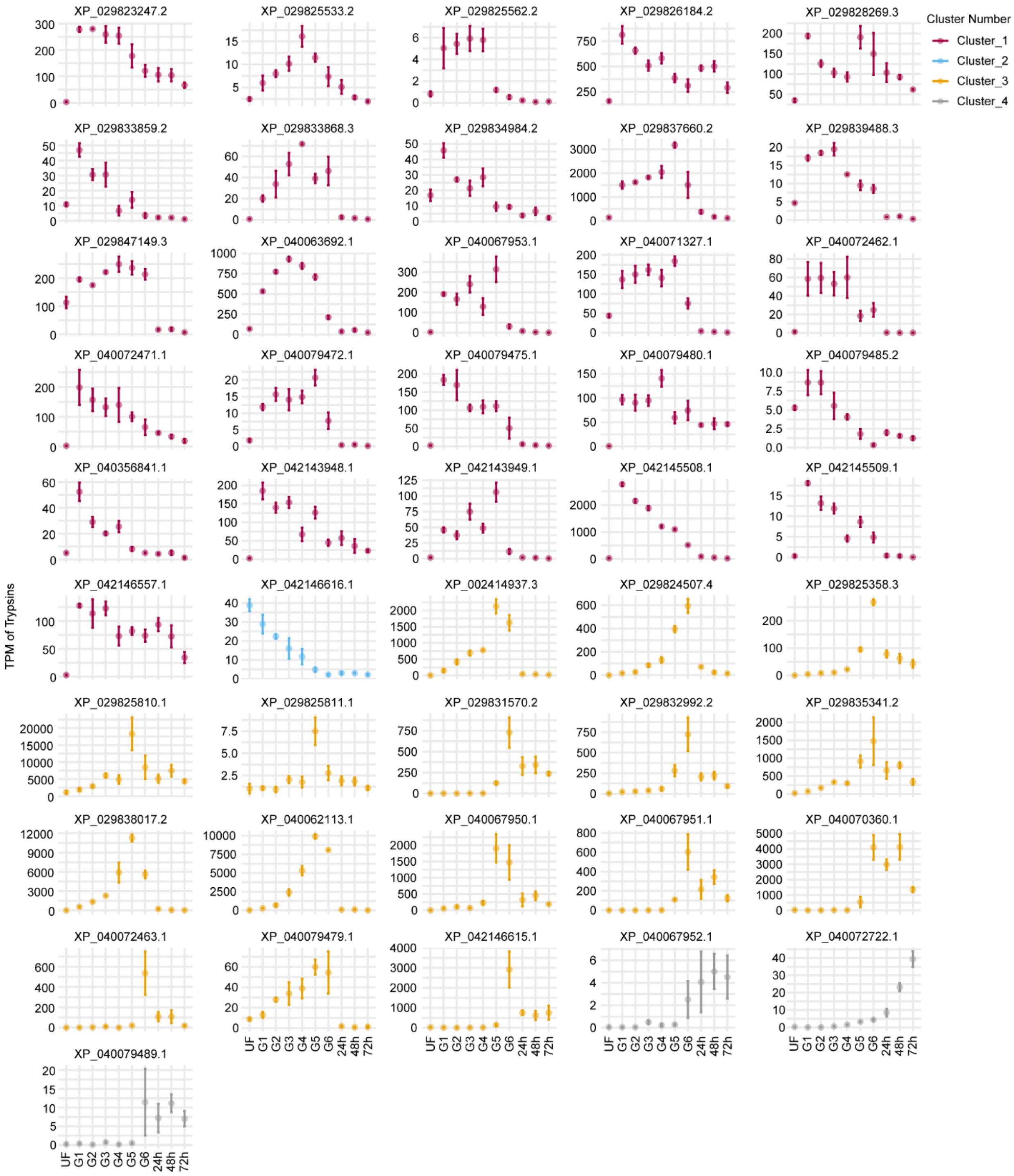

Supplementary file 6: Transcriptional profile of CDS coding for putative trypsin-like peptidases. Colors represent transcripts found in cluster 1 (purple), cluster 2 (blue), cluster 3 (yellow), or cluster 4 (gray). The dots represent the average TPM value found in each feeding stage, and the error bars represent the standard deviation of the mean.

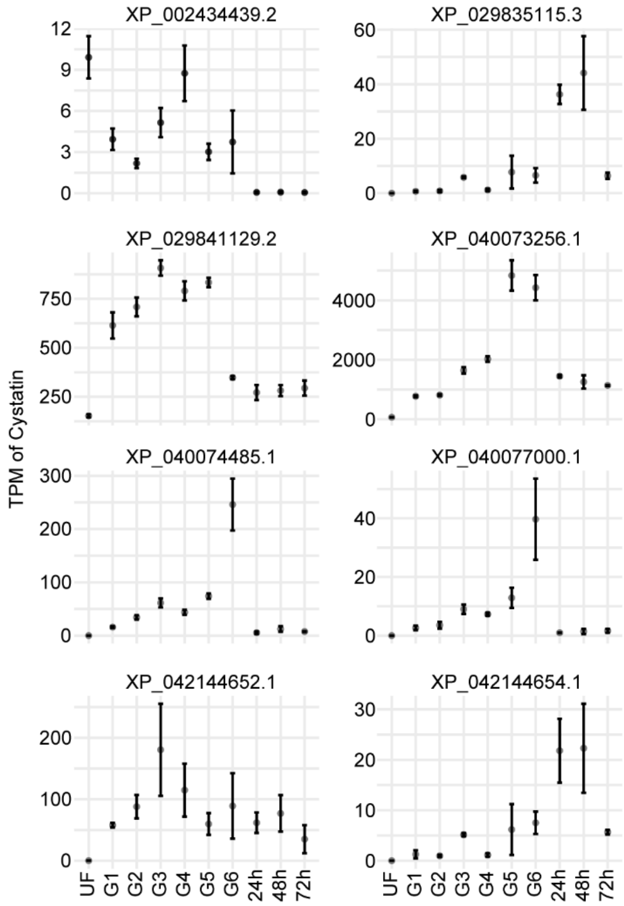

Supplementary Figure 7: Transcriptional profile of CDS coding for putative cystatins at different feeding stages. The dots represent the average TPM value found in each feeding stage, and the error bars represent the standard deviation of the mean.
