# Supplementary material for: A longitudinal transcriptomic analysis from unfed to post-engorgement midguts of adult female Ixodes scapularis
Source: Sci Rep. 2023 Jul 13;13:11360. doi: 10.1038/s41598-023-38207-5 (PMC10345007; doi:10.1038/s41598-023-38207-5)
Supplement: Supplementary file 2 — Supplementary Information. [file 41598_2023_38207_MOESM2_ESM.pdf]

Supplementary files 1 and 2 can be downloaded as a single .ZIP file from the following link:

https://proj-bip-prod-publicread.s3.amazonaws.com/transcriptome/IsMg_2023/IsGutSupFiles.zip

Supplementary File 1: Windows-compatible hyperlinked Excel file of the 10,080 CDS and their functional annotation.

Supplementary File 2: Excel file containing the list of differentially expressed genes from the pairwise comparison of the 10 biological conditions and their functional annotation.
